# Gliflozines as add-on to Arni in echocardiographic, sarcopenic and oxidative stress parameters in elderly patients with chronic heart failure

**DOI:** 10.1007/s40520-025-03049-w

**Published:** 2025-05-19

**Authors:** Giuseppe Armentaro, Velia Cassano, Marcello Magurno, Carlo Alberto Pastura, Marcello Divino, Giandomenico Severini, Domenico Martire, Sofia Miceli, Raffaele Maio, Elisa Mazza, Tiziana Montalcini, Arturo Pujia, Angela Sciacqua

**Affiliations:** 1 Geriatrics Division, “Renato Dulbecco” University Hospital of Catanzaro, 88100 Catanzaro, Italy; 2 Department of Medical and Surgical Sciences, University Magna Graecia of Catanzaro, 88100 Catanzaro, Italy; 3 Geriatrics Division, Ospedale Civile San Giovanni di Dio, Azienda Sanitaria Provinciale di Crotone, 88900, Crotone, Italy; 4 Department of Clinical and Experimental Medicine, University Magna Grecia, 88100 Catanzaro, Italy; 5 Research Center for the Prevention and Treatment of Metabolic Diseases (CR METDIS), University “Magna Graecia” of Catanzaro, 88100 Catanzaro, Italy

**Keywords:** Sarcopenia, SGLT2i, Heart failure, Cardiac index

## Abstract

**Background:**

Sarcopenia is common in patients with heart failure (HF) and it is frequently associated with other comorbidities. Sarcopenia has been linked to an increased risk of major adverse cardiovascular events (MACE) in HF patients.

**Aims:**

The aim of the present study was to evaluate, in a cohort of older adult’s patients affected by HF with reduced ejection fraction (HFrEF) and sarcopenia, already being treated with sacubitril/valsartan, the effect of add-on therapy with SGLT2i on clinical, functional abilities, muscle performance and effects on quality of life.

**Methods:**

We enrolled 147 outpatients. A simple linear regression analysis was performed to assess the correlation between the change in Cardiac Index (CI) and Short physical performance battery (SPPB) values, expressed as (Δ) between baseline and follow-up (ΔT0-12), and several covariates.

**Results:**

After 12 months of treatment, we observed an improvement in the inflammatory profile, moreover there was a reduction of the oxidative stress (*p* < 0.0001) and platelets activation (*p* < 0.0001) parameters. In addition, there was a significant increase in CI and global longitudinal strain and a statistically significant improvement in cognitive function, as shown by Mini-Mental State examination (MMSE) (*p* < 0.0001) score and SPPB (*p* < 0.0001). Considering ΔCI as dependent variation, Δ8-isoprotane resulted the major predictor, justifying 13.3% of its variation. When ΔSPPB was considered as dependent variable, Δ8-Isoprostane was the main predictor of ΔSPPB, justifying 54.6% of its variation.

**Discussion and conclusions:**

This study demonstrated that the addition of SGLT2i to therapy leads to improvements in echocardiographic and sarcopenia-related parameters and biomarkers of oxidative stress and platelet activation.

## Introduction

According to European Working Group on Sarcopenia in Older People two (EWGSOP2), sarcopenia is defined as a progressive and generalized skeletal muscle disorder associated with increased likelihood of adverse outcomes including falls, fractures, physical disability and mortality [1]. Sarcopenia represents a modifiable condition; a multimodal intervention, comprehensive of physical activity and dietary interventions, appears to be the most effective strategy o attenuate the progressive age-related decline in muscle function and enhance both quality of life and life expectancy [2].

Sarcopenia is common in patients with heart failure (HF) [3] and it is frequently associated with other comorbidities [4, 5]. The SICA-HF study revealed that about 20% of patients with chronic HF (CHF) are sarcopenic, a prevalence higher than that observed in patients without CHF [6]. Sarcopenia could potentially worsen the prognosis observed in patients with HF [1]; in fact, sarcopenia has been linked to an increased risk of all-cause mortality and major adverse cardiovascular events (MACE) in patients with HF [7]. The progression of HF is significantly influenced by changes in both muscle function and composition. The loss of skeletal muscle mass in HF patients develops earlier, regardless of left ventricular ejection fraction (LVEF) [3]. In patients with HF, sarcopenia is manifested also as a decrease in myocardial mass [8]. In this context, it is important to examine the myocardium for the assessment and diagnosis of sarcopenia. Of interest, in the last two decades, the evaluation of myocardial deformation represents one of the most important innovations in the echocardiographic field because it considers many parameters that provide information about myocardial function beyond standard echocardiographic indices [9]. In particular, speckle tracking echocardiography (STE) is a second-level echocardiographic technique that allows semi-automatic quantification of myocardial global and regional deformation, with an early detection of cardiac efficiency impairment.

Recently, sodium-glucose co-transporter two inhibitors (SGLT2i) have emerged as a new class of drugs designed to treat patients with type 2 diabetes mellitus (T2DM), but have also been shown to be protective against HF-related events and cardiovascular (CV) mortality [10]. Randomized-trials (EMPAREG-OUTCOME, DECLARE-TIMI 58, CANVAS, VERTIS-CV) and real-life studies [11] demonstrated beneficial effect on reducing the risk of hospitalization for HF. Based on the results of the DAPA-HF [12] and EMPEROR-REDUCED [13] clinical trials, dapagliflozin and empagliflozin demonstrated to significantly reduce the composite primary endpoint of CV mortality and hospitalization in patients with HF with reduced EF (HFrEF) with and without T2DM compared to placebo (dapagliflozin by 26% and empagliflozin by 25%).

The aim of the present study was to evaluate, in a cohort of elderly patients affected by HFrEF and sarcopenia, already being treated with sacubitril/valsartan (Sac/Val), the effect of add-on therapy for 12 months with SGLT2i on clinical, echocardiographic, laboratory parameters, functional abilities, muscle performance and effects on quality of life.

## Materials and methods

### Study population

In the present study, we enrolled 147 outpatients (131 males and 16 females, mean age 72.5 ± 6.9 years), from 2020 to 2023, afferent to the Geriatric Unit of “Magna Graecia” University Hospital of Catanzaro. All patients presented diagnosis of sarcopenia and HFrEF according to ESC guidelines. At baseline, all enrolled patients were in treatment with Sac/Val for at least 12 months. Exclusion criteria were: chronic kidney disease stage IV K-DOQI (eGFR < 30 ml/min/1.73 m2, CKD-EPI), severe hepatic impairment (Child-Pugh Class C), history of angioedema, previous diagnosis of dementia or serious psychiatric disorders. In accordance with guideline recommendations, patients received Dapagliflozin or Empagliflozin at a dosage of 10 mg/day. Clinical evaluation, administration of tests, laboratory tests, ECG and colour Doppler echocardiogram were conducted at the baseline and after12 months of follow-up.

The protocol was approved by the University Ethics Committee (2022.384), and written informed consent was obtained from all participants to the “MAgna GraecIa evaluation of Comorbidities in patients with Heart Failure (MAGIC-HF)” study (ClinicalTrials.gov identifier: NCT05915364) and by the local Ethics Committee of Calabria Region, Italy (Catanzaro, Italy, document n. 263–23 July 2020). This study met the standards of good clinical practice (GCP) and the principles of the Declaration of Helsinki.

### Study procedures

At the baseline, all patients underwent to an accurate medical history and a complete physical examination with the determination of the main anthropometric and hemodynamic parameters. Relevant comorbidities and the type of drug therapies were also recorded. Evaluation of the NYHA functional class was carried out as suggested by current guidelines [14]. All patients were screened for possible and probable presence of sarcopenia (SARC-F questionnaire [15] and evaluation of muscle strength with the handgrip test (HGS) [16] and muscle quantity (ASSM) [1]. Moreover, patients were submitted to Comprehensive geriatric assessment (CGA) using the follow evaluations scales: Mini-Mental State Examination (MMSE) [17]; Activities of daily living (ADL) [18]; Instrumental Activities of daily living (IADL) [18]. In addition, presence of depressive symptoms was estimated with the Geriatric Depression Scale GDS [19]. Quality of life assessment was performed using the Minnesota Living with Heart Failure Questionnaire (MLHFQ) [20] and Kansas City Cardiomyopathy Questionnaire– Clinical Summary (KCCQ-QS), Kansas City Cardiomyopathy Questionnaire- Overall Score (KCCQ-OS) [21]. We determined the condition of sarcopenia with BIA and evaluated of the severity of the condition through Short physical performance battery (SPPB).

All patients underwent a 12- lead electrocardiogram (ECG), blood chemistry tests and a full echocardiogram-colour-Doppler.

The evaluation of clinical Blood Pressure (BP) was performed according to current guidelines. Measurements of BP were acquired in the left arm of patients in sitting position using a semi-automatic sphygmomanometer (OMRON, M7 Intelli IT) after five min of rest. BP values were the average of three measurements. This evaluation was repeated on three different occasions at least 2 weeks apart. Subjects with a clinic SBP > 140 mmHg and/or DBP > 90 mmHg were defined as hypertensive [13]. Pulse pressure (PP) values were acquired as the difference between systolic and diastolic BP measurements.

### Echocardiographic parameters

Echocardiographic recordings were performed using a VIVID E-95 ultrasound system (GE Technologies, Milwaukee, Wisconsin, USA) with a 2.5 MHz transducer. All patients were examined at rest and in the left lateral decubitus position. Measurements were obtained according to the recommendations of the American Society of Echocardiography [22]. To minimize measurement errors, the echocardiographic examinations were carried out by the same expert operator who, moreover, was not aware of the patient’s clinical data; the values ​​considered represent the average of at least three measurements. Left ventricular mass (LVM) was calculated using the formula proposed by Devereux and corrected for body surface area (BSA), to derive the LVM index (LVMI) [23]. Among the parameters of left ventricular global systolic function, left ventricular ejection fraction (LVEF) and cardiac index (CI) were evaluated [22]. LVEF was calculated by the Simpson biplane method. Right ventricular systolic parameters were also measured, by estimating the systolic pulmonary arterial pressure (S-PAP) [24].

The diameter of the right ventricular outflow tract (RVOT) and the right atrium area (RAA) were obtained according to ASE recommendations [22]. The movement of the tricuspid annulus was recorded at the free wall of the RV for the tricuspid annular plane systolic excursion (TAPSE), which expresses the right longitudinal function. In addition, for a more complete assessment of right ventricular function, the TAPSE/S-PAP ratio, an index of the right ventricular length/strength relationship.

A 2D speckle tracking analysis was retrospectively performed using vendor-specific 2D speckle tracking software (EchoPAC PC, version 113.0.5, GE Healthcare, Horten, Norway). Manual tracings of the endocardial border during end-systole in three apical views was performed to evaluate global longitudinal strain (GLS) [25].

### Laboratory parameters

After at least 12 h fasting, the laboratory measurements were performed. The glucose oxidation method (Beckman Glucose Analyzer II; Beckman Instruments, Milan, Italy) was utilized to measure plasma glucose, and a chemiluminescence-based assay (Roche Diagnostics) for plasma insulin determination. Insulin sensitivity was determined with the metabolic homeostasis method (Homeostasis Model Assessment of Insulin Resistance, HOMA). An enzymatic method (Roche Di-agnostics GmbH, Mannheim, Germany) was used to detect total, low and high-density lipoprotein (LDL, HDL) cholesterol and triglyceride concentrations. Serum creatinine was determined using a Roche Creatinine Plus assay (Hoffman-La Roche, Basel, Switzerland) on a clinical chemistry analyser (Roche/Hitachi Modular Analytics System, P Module). Renal function was evaluated by calculating the estimate glomerular filtration rate (e-GFR) using the CDK-EPI equation [26]. An enzyme-linked immunosorbent assay (Elecsys proBNP assay, Roche Diagnostics) was utilized to assess N-terminal pro-brain natriuretic peptide (NT-proBNP) levels. Serum sodium and potassium levels were obtained by indirect potentiometry (Cobas, Roche) and high sensitive C-reactive protein (hs-CRP) by an automated instrument (Cardio-Phase1hsCRP), Milan, Italy).

For the analysis of oxidative stress (8-isoprostane and Nox-2) and platelets activation (Glycoprotein- VI and sP-selectin) biomarkers, blood samples were collected in tubes with separator gel and centrifuged at 4,000 rpm for 15 min to obtain serum samples. Quantitative determinations of the 8-isoprostane (ELISA kit Cayman Chemical, Ann Arbor, MI, USA), Nox-2 (ELISA kit MyBioSource, San Diego, CA, USA), human glycoprotein VI (GPVI) and Sp-selectin (all from ELISA kit MyBioSource, California, United States), were performed with commercial ELISA immunoassays according to the manufacturer’s instructions.

### Statistical analysis

Continuous variables are expressed as mean and standard deviation (SD) (normally distributed data) or as the median and interquartile range (IQR) (non-normally distributed data). Categorical data are expressed as numbers and percentages. The evolution of therapies over time was assessed with the χ2 test. Longitudinal changes in key variables at follow-up were analysed with the t-test or Wilcoxon’s test for paired data, and comparisons between the two groups were made with the t-test and Mann–Whitney test for unpaired data when appropriate. A simple linear regression analysis was performed to assess the correlation between the change in CI and SPPB values, expressed as (Δ) between baseline and follow-up (ΔT0-12), and the change in several covariates, also expressed as ΔT0-12. Variables that reached statistical significance were entered into a stepwise multivariate linear regression model to assess the magnitude of their individual effects on ΔCI and ΔSPPB. Differences were considered significant at *p* < 0.05. Statistical analysis was carried out using the SPSS V20.0 program or Windows (SPSS Inc., Chicago, IL, USA).

## Results

The study population included 131 males (89.1%) and 16 females (11.9%) with a mean age of 72.5 ± 6.9 years. All subjects in the study population were diagnosed with sarcopenia according to the criteria of the European Working Group on Sarcopenia in Older People (EWGSOP2) (36). All patients had HFrEF. Among these, 7 patients had HF mild preserved EF (HFmpEF); taking into account associated comorbidities, 42.6% of patients had ischaemic heart disease, 20.4% had atrial fibrillation (AF), 39.4% had TD2M, 47.6% had chronic pulmonary obstructive disease (COPD) (Table 1).

**Table 1:** Comorbidities and pharmacotherapy of study population at baseline according to sarcopenia diagnosis

	Whole population (*n*. 147)
Male gender, *n (%)*	131 (89.1)
Age **≥** 70	61 (41.5)
IHD, *n (%)*	68 (46.2)
VHD, *n (%)*	37 (25.1)
Arterial hypertension, *n (%)*	98 (66.6)
AF, *n (%)*	30 (56.6)
HFimpEF *n (%)*	7 (4.7)
Cerebral vasculopathy, *n (%)*	30 (20.4)
PAD, *n (%)*	15 (10.2)
Dislipidemia, *n (%)*	120 (81.6)
SAS, *n (%)*	64 (43.5)
CKD, *n (%)*	47 (31.9)
COPD, *n (%)*	69 (47.6)
NAFLD, *n (%)*	48 (32.6)
T2DM, *n (%)*	58 (39.4)
ICD-CRT-D, *n (%)*	41 (27.9)
Smokers, *n (%)*	103 (70.0)
β-blockers, *n (%)*	146 (99.3)
ACEi/ARBs, *n (%)*	7 (4.7)
MRAs, *n (%)*	100 (68.0)
ARNI, *n (%)*	147 (100.0)
GLP-1RA, *n (%)*	162 (47.6)
Insulin, *n (%)*	25 (17.1)
Statins, *n (%)*	114 (77.5)
Diuretics, *n (%)*	147 (100.0)
Antiplatlets drug, *n (%)*	85 (57.8)
OAC, *n (%****)***	24 (13.3)
OADs, *n (%)*	78 (53.1)

IHD: ischemic heart disease; HFimpEF: Heart Failure with improved Ejection Fraction; VHD: Valvular heart disease; AF: atrial fibrillation; PAD: Peripheral Artery Disease; SAS: sleep apnea syndrome; CKD: Chronic kidney disease; COPD: Chronic obstructive pulmonary disease; NAFLD: Non-alcoholic fatty liver diasease; T2DM: type 2 diabetes mellitus; ICD-CRT-D: Implantable Cardioverter Defibrillator - Cardiac Resynchronization Therapy Defibrillator; ACEi: Angiotensin-converting enzyme inhibitors; ARBs: Angiotensin II receptor blockers; MRAs: mineralocorticoid receptor antagonists; SGLT2i: sodium-glucose cotransporter 2 inhibitors; ARNI: angiotensin receptor/neprilysin inhibitor; GLP-1 RA: Glucagon-like-peptide 1 receptor agonists; OAC: oral anticoagulant; OADs: oral antidiabetic drug

At baseline, 100% of patients were treated with sac/val therapy. At baseline, patients had high circulating levels of NT-ProBNP, oxidative stress and platelet activation biomarkers. In the entire population, a significant improvement in haemodynamic and clinical parameters was observed after 12 months of follow-up, heart rate (80.3 ± 2.5 vs. 74.1 ± 4.6 bpm, *p* < 0.0001) (Table 2).

**Table 2:** Comparison of baseline and follow-up according to the diagnosis of sarcopenia on clinical, haemodynamic and laboratory parameters and functional tests

	Baseline	Follow up	*p*
SARC F	6.8 ± 2.0	3.3 ± 2.1	< 0.0001
HG, *kg*	18.1 ± 5.1	20.9 ± 4.1	< 0.0001
ASMM, *kg/m²*	14.6 ± 3.6	14.8 ± 4.1	0.045
SPPB, *pt*	5.6 ± 1.7	7.1 ± 1.9	< 0.0001
GDS, *pt*	11.6 ± 2.7	6.6 ± 1.6	< 0.0001
GAIT Speed, *m/s*	0.8 ± 0.1	1.0 ± 0.1	< 0.0001
MLHFQ	90.0 ± 3.7	80.5 ± 4.3	< 0.0001
KCCQ-CS, *pt*	60.7 ± 1.5	64.2 ± 1.5	< 0.0001
KCCQ-OS, *pt*	62.5 ± 1.3	64.2 ± 1.3	< 0.0001
BMI, *kg/m2*	17.7 ± 2.5	19.7 ± 2.7	< 0.0001
SBP, *mmHg*	120.9 ± 12.3	117.5 ± 10.5	< 0.0001
**D**BP, *mmHg*	74.0 ± 7.8	69.1 ± 6.6	< 0.0001
HR, *bfm*	80.3 ± 2.5	74.1 ± 4.6	< 0.0001
PP, *mmHg*	46.8 ± 15	48.4 ± 12.42	0.227
HCT (%)	33.9 ± 1.1	36.91 ± 1.3	< 0.0001
Hb, *g/dl*	10.0 ± 0.7	10.9 ± 0.8	< 0.0001
PLT, *103/mm3*	200.1 ± 52.2	196.3 ± 50.6	0.366
Na, *mmol/l*	140.7 ± 2	138.8 ± 1.5	< 0.0001
K, *mmol/l*	4.4 ± 0.4	4.7 ± 0.4	< 0.0001
HOMA, *pt*	18.4 ± 3.8	16.5 ± 3.8	< 0.0001
Albumin, *mg/dl*	3.7 ± 0.3	4.9 ± 0.3	< 0.0001
Vitamin D, *ng/ml*	10.22 ± 3.3	21.9 ± 7.9	< 0.0001
Creatinine, *mg/dl*	1.7 ± 0.2	1.5 ± 0.2	< 0.0001
*eGFR*, *ml/min*	40.3 ± 5.9	50.1 ± 8.6	< 0.0001
Total protein, *g/dl*	5.7 ± 0.3	6.9 ± 0.4	< 0.0001
PLT, *103/mm3*	200.1 ± 52.2	196.3 ± 50.6	0.366
LDL, *mg/dl*	80.8 ± 34.2	67.8 ± 34.0	< 0.0001
HDL, *mg/dl*	27.1 ± 4.0	40.0 ± 6.7	< 0.0001
Triglycerides, *mg/dl*	70.4 ± 10.4	77.4 ± 21.7	< 0.0001
8-isoprostane *(pg/ml)*	72.4 ± 9.7	55.3 ± 9.4	< 0.0001
sP-selectine, *(ng/ml)*	118.3 ± 19	100 ± 16.2	< 0.0001
GPVI *(pg/ml)*	62.5 ± 7.6	52.4 ± 7.7	< 0.0001
Nox − 2 *(nmol/L)*	1.0 ± 0.2	0.8 ± 0.1	< 0.0001
hs-CRP *(mg/L)*	5.4 ± 0.7	3.6 ± 0.7	< 0.0001
Uric acid *(mg/dl)*	7.5 ± 0.7	5.3 ± 1.3	< 0.0001
NT-pro-BNP*(pg/ml)*	1786.8 ± 115.8	1362.1 ± 211.8	< 0.0001
MMSE	22.1 ± 2.4	24.1 ± 2.6	< 0.0001
ADL	3.5 ± 0.8	4.5 ± 1.0	< 0.0001
IADL	4.4 ± 0.9	5.4 ± 0.7	< 0.0001

HG: handgrip; SMI: Skeletal Mass Index; ASMM: Appendicular Skeletal Muscle Mass; SPPB: short physical performance battery; MLHFQ: Minnesota Living with Heart Failure Questionnaire; GDS: Geriatric Depression Scale; KCCQ-CS: Kansas City Cardiomyopathy Questionnaire– Clinical Summary; KCCQ-OS: Kansas City Cardiomyopathy Questionnaire- Overall Score; BMI: Body mass index; SBP: Systolic blood pressure, DBP: Diastolic blood pressure; HR: heart rate; Hct: Hb: hemoglobin; PLT: platelet count; Na: Sodium; K: Potassium; HOMA: Homeostatic Model eGFR: estimate glomerular filtration rate; LDL: low density lipoproteins; HDL: High-Density Lipoprotein; NT-pro-BNP: N-terminal pro-brain natriuretic peptide; GPVI: Glycoprotein VI; hs-CRP: high sensitivity C reactive protein; MMSE: Mini- Mental State Examination; ADL: Activities of daily living; IADL: Instrumental Activities of daily living

**Fig. 1:**
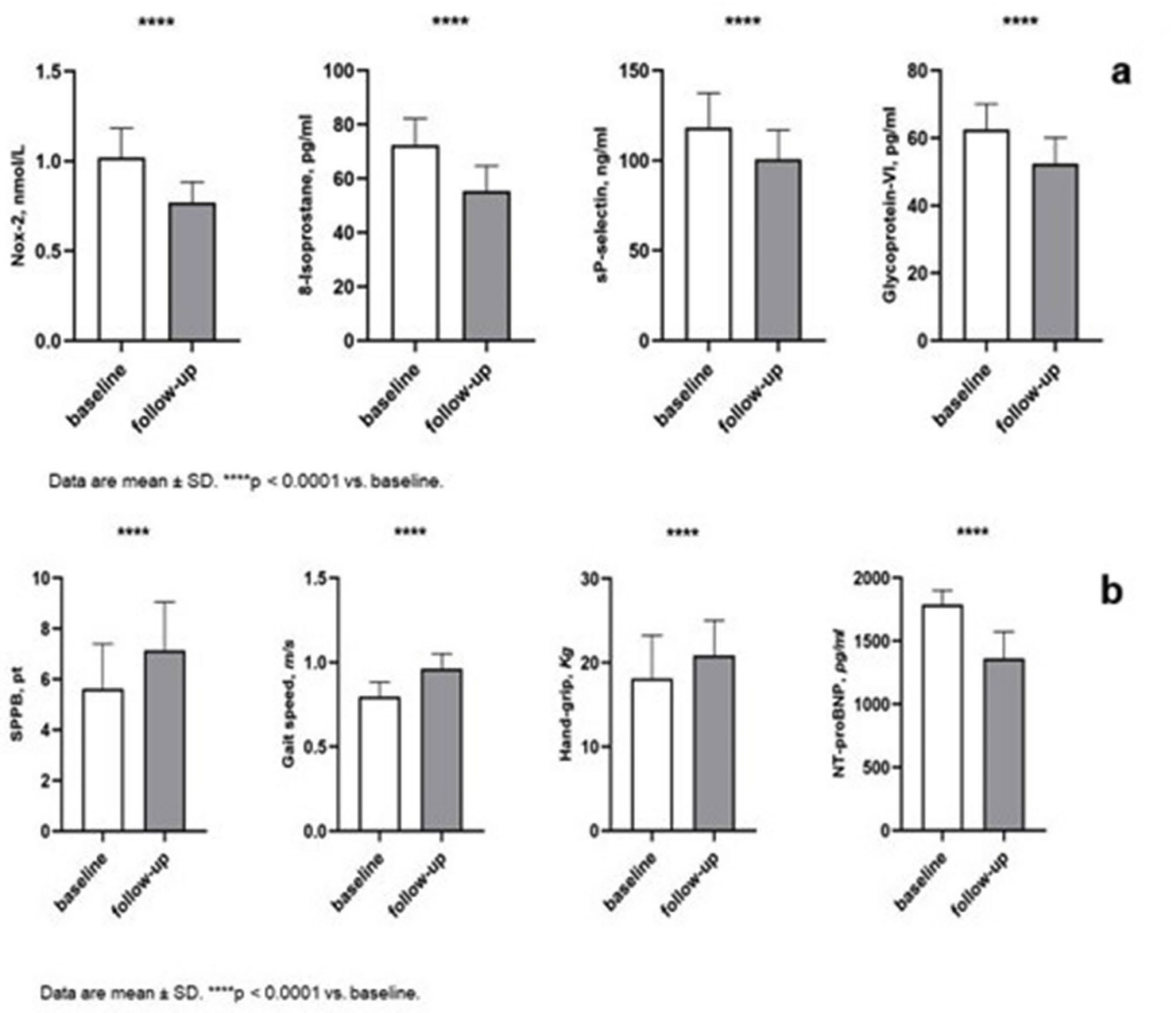
Comparison between baseline and follow-up between biomarkers of oxidative stress and platelet activation. Data are mean ± SD. ****p < 0.0001 vs. baseline (**A**). Comparison between baseline and follow-up between markers of functional abilities and NT-proBNP. Data are mean ± SD. ****p < 0.0001 vs. baseline (**B**)

After 12 months of treatment with SGLT2i as add-on to Arni, we observed an improvement in the inflammatory profile, as reflected by the reduction in the levels of hs-CRP (5.4 ± 0.7 vs. 3.6 ± 0.7 mg/l, *p* < 0.0001) and uric acid (7.5 ± 0.7 vs. 5.3 ± 1.3 mg/dl, *p* < 0.0001); moreover there was a reduction of the oxidative stress parameters Nox-2 (1.0 ± 0.2 vs. 0.8 ± 0.1 nmol/l, *p* < 0.0001) and 8-Isoprostane (72.4 ± 9.7 vs. 55.3 ± 9.4 pg/ml, *p* < 0.0001) and platelets activation biomarkers sP-Selectin (118.3 ± 19.0 vs. 100.0 ± 16.2 ng/ml (*p* < 0.0001) and glycoprotein-VI (GPVI) (62.5 ± 7.6 vs. 52.4 ± 7.7 pg/ml, *p* < 0.0001) (Fig. 1A).

Moreover, at the follow-up, there was an improvement in hemodynamic compensation as evidenced by the reduction in circulating NT-proBNP levels (1786.8 ± 115.8 vs. 1362.1 ± 211.8 pg/ml, *p* < 0.0001) and an improvement in renal function, as evidenced by eGFR (40.3 ± 5.9 vs. 50.1 ± 8.6 ml/min/1.73m2, *p* < 0.0001).

Table 3 shows the echocardiographic characteristics of the entire study population.

**Table 3:** Comparison of baseline and follow-up according to the diagnosis of sarcopenia on echocardiographic parameters

	Baseline	Follow up	*p*
LAVi, *ml/m*^*2*^	40.3 ± 4.5	33.3 ± 5.3	< 0.0001
LVEDV/BSA, *ml/m²*	65.3 ± 3.6	58.5 ± 4.3	< 0.0001
LVESV/BSA, *ml/m²*	54.6 ± 7	41.7 ± 3.7	< 0.0001
LVEF, *%*	33.0 ± 5.3	36.8 ± 5.3	< 0.0001
CI, *ml/min/1.73 m*^*2*^	1685.1 ± 200	1956 ± 205.6	< 0.0001
E/A	0.6 ± 0.1	0.7 ± 0.1	< 0.0001
E/e’	17.8 ± 3.7	16.0 ± 3.7	< 0.0001
GLS, *%*	-8.2 ± 1.4	-11.2 ± 1.8	< 0.0001
RVOTp, *m/s*	2.8 ± 0.5	2.1 ± 0.3	< 0.0001
Right Atrial Area, *cm*^*2*^	20.8 ± 0.5	17.4 ± 2.6	< 0.0001
TAPSE, *mm*	16.2 ± 1.7	17.6 ± 1.6	< 0.0001
s-PAP, *mmHg*	34.3 ± 7.8	27.8 ± 8.0	< 0.0001
IVC, *mm*	18.8 ± 2.0	16.1 ± 2.1	< 0.0001
TAPSE/s-PAP, *mm/mmHg*	16.2 ± 1.4	17.6 ± 1.6	< 0.0001

LAVi: left atrial volume index; LVESV/BSA: Left Ventricular End-Systolic Volume indexed to Body Surface Area; LVEDV/BSA: Left Ventricular End-Diastolic Volume indexed to Body Surface Area; LVEF: Left ventricular Ejection Fraction; CI Cardiac Index; E/A: ratio between wave E (the wave of rapid filling in early diastole) and wave A (the wave of atrial contraction); E/e’: between wave E and wave e′ (reliable estimate of changes in end-diastolic blood pressure); GLS: global longitudinal strain; RVOTp: Right Ventricular Outflow Tract proximal; TAPSE: Tricuspid annular plane systolic excursion; s-PAP: systolic pulmonary arterial pressure; IVC: Inferior Vena Cava

At the follow-up, there was a significant reduction in left cavitary diameters with a statistically significant increase in LVEF (33.0 ± 5.3 vs 36.8 ± 5.3%, p < 0.0001), CI (1685.1 ± 200.0 vs 1956.0 ± 205.6 ml/min/1.73 m2, p < 0.0001), GLS (-8.2 ± 1.5 to -10.9 ± 1.3%, p < 0.0001) and increase in right function indices, in fact, we observed an increase in TAPSE (16.2 ± 1.7 vs 17.6 ± 1.6 mm, p < 0.0001). In addition, there was an improvement in diastolic function with a reduction in left ventricular filling pressures as evidenced by a reduction in E/e’ ratio (17.8 ± 3.7 vs. 15.0 ± 3.7 *p* < 0.0001) and systemic congestion, as evidenced by inferior Vena Cava diameter (IVC) (18.8 ± 2.0 vs. 17.6 ± 1.6 mm, *p* < 0.0001).

Regarding the scales used in the Geriatric Multidimensional Assessment, after 12 months treatment with SGLT2i, there was a statistically significant improvement in cognitive function, as shown by the increase in the MMSE score (22.1 ± 2.4 vs. 24.1 ± 2.6 pt, *p* < 0.0001), contextually there was an improvement in functional ability as evidenced by SPPB (5.6 ± 1.7 pt vs. 7.1 ± 1.9 pt, *p* < 0.0001), GAIT Speed (0.8 ± 0.1 m/sec vs. 1.0 ± 0.1, *p* < 0.0001), IADL (4.4 ± 0.9 vs. 5.4 ± 0.7, *p* < 0.0001). In addition, we observed an improvement in quality of life as demonstrated by MLHFQ (90.0 ± 3.7 vs. 80.5 ± 4.3 pt, *p* < 0.0001), KCCQ-CS (60.7 ± 1.5 vs. 64.2 ± 1.5 pt, *p* < 0.0001), KCCQ-OS (62.5 ± 1.3 pt vs. 64.2 ± 1.3 pt, *p* < 0.0001) and GDS score (11.6 ± 2.7 vs. 6.6 ± 1.6 pt, *p* < 0.0001) (Fig. 1B).

A simple linear regression analysis was performed to assess the correlation ΔCI, ΔSPPB and different covariates (Table 4). ΔCI was significantly and indirectly correlated with Δ8-isoprostane (*r*= -0.570, *p* < 0.0001), ΔsP-selectin (*r*= -0.396, *p* < 0.0001), Δhs-CRP (*r*= -0.242, *p* = 0.006). Subsequently ΔSPPB was significant and indirectly correlated with ΔNox-2 (*r*= -0.232, *p* < 0.0001), Δ8-isoprostane (*r*= -0.453, *p* < 0.0001), ΔsP-selectin (*r*= -0.290, *p* = 0.012) and age (*r*= -0.125, *p* = 0.030).

**Table 4:** Simple linear regression between ΔCI, ΔSPPB and different covariates in the study population

	ΔCI	Δ SPPB
	r/p	r/p
Δ hs-CRP, *mg/l*	-0.242/0.006	-0.009/0.884
Δ Nox-2, *nmol/l*	-0.018/0.831	-0.232/<0.0001
Δ 8-Isoprostane, *pg/ml*	-0.570/<0.0001	-0.453/<0.0001
Δ sP-Selectine, *ng/ml*	-0.396/<0.0001	-0.290/0.012
Δ Gp-VI, *pg/ml*	-0.012/0.877	-0.046/0.401
Δ HOMA	-0.069/0.355	-0.070/0.203
Δ CI, *ml/min/1.73 m*^*2*^	--	0.028/0.660
Age, *1 years*	-0.045/0.592	-0.125/0.030
Male sex, *yes/no*	0.081/0.273	-0.029/0.600
Δ Handgrip, *Kg*	0.110/ 0.166	--
Δ gait speed, *m/s*	0.092/0.210	--
Δ eGFR, *ml/min/1.73 m2*	0.076/ 0.486	--
Δ NT-proBNP, *pg/ml*	0.123/0.553	--

hs-CRP, highly sensitive c-reactive protein; Nox-2: NAPDH Oxidase 2; Gp-VI, Glycoprotein-VI; HOMA, homeostatic model assessment; eGFR, estimated glomerular filtration rate; SPPB: Short physical performance battery; CI: Cardiac index

Variables reaching statistical significance were introduced in a stepwise multivariate linear regression model to identify the independent predictors of ΔCI and ΔSPPB (Table 5).

**Table 5:** Multivariate linear regression between ΔCI (a), ΔSPPB (b) and different covariates in the study population **a)**

Δ CI	*R*^2^ partial	*R*^2^ total	*p*
Δ 8-Isoprostane, *pg/ml*	13.3%	13.3%	< 0.0001
Δ sP-Selectin, *ng/ml*	11.1%	24.4%	< 0.0001
Δ hs-CRP, *mg/l*	4.0%	28.4%	0.005
**Δ SPPB**	***R*** ^2^ **partial**	***R*** ^2^ **total**	***p***
**Δ 8-Isoprostane**, *pg/ml*	54.6%	54.6%	< 0.0001
**Δ Nox-2**, *nmol/l*	3.6%	58.2%	0.001
**Δ sP-Selectine**	1.5%	59.7%	0.022
**Age**, *years*	1.3%	61.0%	0.029

SPPB: Short physical performance battery; hs-CRP, highly sensitive c-reactive protein; Nox-2: NAPDH Oxidase 2; HOMA, homeostatic model assessment; CI: Cardiac index

Δ8-isoprotane was the major predictor of ΔCI accounting for 13.3% of its variation, ΔsP-selectin and Δhs-CRP added respectively another 11.1% and 4.0%. Moreover, Δ8-Isoprostane was the main predictor of ΔSPPB, justifying 54.6% of its variation, ΔNox-2, ΔsP-selectin and age added respectively another 3.6%, 1.5% and 1.3%.

## Discussion

This study, conducted in older adults’ outpatients with HFrEF stratified by diagnosis of sarcopenia, demonstrated how optimization of medical treatment was associated with improvement in clinical, hemodynamic and functional abilities. It’s known that ageing is a major risk factor for HF and is associated with physiological changes, including increased chronic inflammation and oxidative stress (inflammaging), increased myocardial fibrosis, vascular stiffness, reduced renal function, peripheral and respiratory skeletal muscle dysfunction, autonomic dysfunction and metabolic adaptations, malnutrition, all of which contribute to reduced exercise and functional capacity in older adults [27].

In this study, we observed that the introduction of SGLT2i in patients in therapy with Sac/Val, improved echocardiographic parameters. The SGLT2i treatment was associated with reduction in left atrial volume index (LAVI) and end-systolic and end-diastolic LV volumes, together with this also diastolic function parameters were improved, as evidenced by E/A ratio increase and E/e’ reduction. At the follow-up, we observed also an improvement in LV contractility as demonstrated by the significant change in GLS values and LVEF.

Moreover, after 12 months of treatment with SGLT2i, there was an improvement also in parameters and inflammation, as well as biomarkers of oxidative stress. Inflammation and oxidative stress are two interrelated processes that play an important role in the development of HF. Oxidative stress occurs when the body’s antioxidant defenses are overwhelmed by the production of reactive oxygen species (ROS). In the heart, an excess of ROS can lead to the development and progression of maladaptive myocardial remodelling [28]. ROS production in the heart is primarily achieved by the mitochondria, enzyme nicotinamide adenine dinucleotide phosphate (NADPH) oxidases, xanthine oxidase, and uncoupled nitric oxide synthase (NOS). Under pathological conditions, the electron transport chain of the mitochondria induces the formation of large quantities of superoxide. This increase has been shown to contribute to cardiomyocyte damage and larger myocardial injury. A previous study conducted by our group demonstrated the six-months treatment with Sac/Val reduced oxidative stress levels in HF patients [29]. The reduction in oxidative stress levels may be due to the ability of Sac/Val to block the angiotensin II receptor and the reduction in inflammatory indices. SGLT2i have also been shown to reduce oxidative stress by increasing the production of antioxidants such as glutathione and reducing the production of ROS [30, 31]. This reduction in oxidative stress may contribute to the beneficial effects of SGLT2i on CV outcomes and may justify the improvement in strength parameters. Therefore, a 12-month treatment with SGLT2i as an add-on could have a synergistic effect on oxidative stress in HFrEF patients.

Of interest, in this study, we demonstrated that the introduction of SGLT2i in patients in therapy with Sac/Val, improved also parameters related to sarcopenia. This study demonstrated how the synergistic effect of Sac/Val and SGLT2i therapy was beneficial in improving strength as measured by handgrip test parameters, gait speed, SPPB, and consequently quality of life.

There is limited evidence directly linking Sac/Val to improvements in sarcopenia in HF patients; a recent multicenter trial conducted by Nugara et al. demonstrated that Sac/Val improved cardiopulmonary exercise capacity in patients with HFrEF [32]. This improvement in exercise capacity is often associated with better overall physical performance, but does not specifically measure changes in skeletal muscle strength or mass compared to other treatments such as enalapril. While Sac/Val has been shown to be superior to enalapril in reducing the risk of death and hospitalization for HF, studies do not specifically address its comparative effects on skeletal muscle. To date, the focus has largely been on cardiac outcomes, such as reductions in myocardial fibrosis and improvements in cardiac function, rather than direct assessments of muscle strength. The EMPA-ELDERLY study - a randomized, double-blind, placebo-controlled, 52-week clinical trial - showed that empagliflozin was effective in reducing body weight without affecting muscle mass or strength in older adults with T2DM [33]. Similar results were seen in another study of patients with T2DM treated with dapagliflozin [34]. However, studies of the effect of SGLT2 inhibitors on skeletal muscle mass, strength and exercise capacity in patients with HF are lacking; a recent meta-analysis showed that SGLT2 inhibitors may improve health-related quality of life (HRQoL) and exercise capacity in patients with CHF, which could lead to an increase in physical activity with benefits for muscle mass [35].

Data from this study showed also significant reduction platelets activation biomarkers, such as sP-selectin and GPVI levels after introducing SGLT2i over a 12-month follow-up period. Platelets play an important role in skeletal muscle regeneration and sarcopenia [36]. Several studies have found associations between platelet count, platelet-lymphocyte ratio (PLR) and sarcopenia. A study by Fang-Yih Liaw et al. showed that higher platelet counts and PLR were associated with an increased risk of sarcopenia in older adults, even after adjustment for confounders [36, 37]. Platelet counts were more strongly associated with handgrip strength and muscle mass than with other sarcopenia parameters [36].

Several studies demonstrated that SGLT2i affect platelet activation and reactivity, suggesting potential benefits for cardiovascular health, particularly in patients with diabetes. A study by Seecheran et al. found that treatment with dapagliflozin significantly reduced platelet reactivity, as evidenced by a 20% reduction in P2Y12 reaction units (PRUs), in patients with type 2 diabetes and stable coronary artery disease. This suggests that dapagliflozin may have an antiplatelet effect, which could be beneficial in reducing the CV risks associated with high platelet activation in diabetic patients [38]. Similar findings were observed with empagliflozin, where it was noted that SGLT2 inhibition may attenuate platelet reactivity through multiple pathways, including improved glycemic control, reduction of dyslipidemia and reduced oxidative stress [11].

Sac/Val and SGLT2i are therapeutic agents with pleiotropic effects on metabolic regulation and reduction of CV and renal complications, as demonstrated in several studies.

To date, this is the first study to be conducted in an older adult’s population with HFrEF and sarcopenia in which the benefit of the addition of SGLT2i to therapy has been demonstrated on clinical, hemodynamic and echocardiographic parameters. Of particular interest, this study shows how the addition of SGLT2i to therapy leads to improvements in echocardiographic and sarcopenia-related parameters and biomarkers of oxidative stress and platelet activation.

Further studies are needed to support the hypothesis that they may play an important role in a complex pathology such as sarcopenia. In any case, the improvement in symptoms and quality of life is very important due to the improvements in hemodynamic, biochemical and echocardiographic parameters seen with Sac/Val and SGLT2i. However, further studies with longer follow-up are needed to better elucidate the effects of optimal HF treatment on sarcopenia.

## Data Availability

No datasets were generated or analysed during the current study.

## Declarations

### Data sharing

The study is registered at ClinicalTrials.gov **(**NCT05915364). Researchers wishing to access data should contact the corresponding author.
